# Do participants in major, practice-changing breast cancer trials reflect the breast cancer patient population?

**DOI:** 10.1186/1745-6215-14-S1-P33

**Published:** 2013-11-29

**Authors:** Shaun Treweek, Ruth Dryden, Colin McCowan, Alison Harrow, Alastair Thompson

**Affiliations:** 1 University of Aberdeen, Aberdeen, UK; 2 University of Dundee, Dundee, UK; 3 University of Glasgow, Glasgow, UK

## Background

Inadequate consideration of applicability is the most frequent criticism by clinicians of randomised trials, especially regarding narrow eligibility criteria.

## Aim

To calculate how many women diagnosed with breast cancer between 1993 and 2008 in Tayside, Scotland, would have been eligible to participate in 12 major breast cancer trials.

## Methods

Major trials were identified by reviewing phase III trials of adjuvant treatment referenced in recent national guidelines. This list was shortened by delegates at a meeting of the Scottish Cancer Trials Breast Group. Inclusion criteria for these trials were extracted from trial protocols and applied to women in the 16-year Tayside breast cancer dataset.

## Results

33 breast cancer specialists ranked 39 trials in order of importance with the 12 most selected included in this study. Of 4811 women in the Tayside dataset, the number meeting eligibility criteria for the 12 trials ranged from 822 (17%) to 3419 (71%). Reasons for exclusion included age and comorbidity. Many women received a treatment evaluated in a trial but would themselves not have been eligible for the trial. For example, of 572 patients prescribed anastrazole and tamoxifen, only 281 were eligible for the trial evaluating that combination. Work to explicitly compare treatment decisions in clinical practice to trial eligibility criteria is ongoing.

## Conclusions

Trialists should make their trials as widely applicable as possible. This may not have been the case for some major breast cancer trials, where women in the community receiving an intervention would not themselves have been eligible for the trial.

